# The Prevention of Venereal Disease

**Published:** 1917-03-03

**Authors:** H. Bryan Donkin


### THE EDITOR'S LETTER-BOX.

### The Prevention of Venereal Disease.

*To the Editor of* The Hospital.

Sir,—I have read with interest the article on this subject which appeared in The Hospital of February 24. No more complete and closely-reasoned reply could be given to the passive, but none the less considerable, antagonism to spreading the knowledge of effectual preventive measures against venereal infections which has been recently evidenced by the attitude of many who have been active in securing State aid towards expediting the cure of these diseases after they have been contracted.

Surely the most obvious methods of checking the spread of venereal infection at its very source are the use of these preventive measures and the restriction by all possible means of the public "soliciting" now being carried on without hindrance by the many congregations of women who in London and other cities, and especially at the railway stations, are permitted to flock around the soldiers of the British Empire. There is great reason to believe that the alleged increase of venereal disease in our Army is due to infection, not imported from abroad, but exported from England by new drafts of men, and by others returning from leave.

With regard to the question of establishing police measures directed against "soliciting," there is doubtless some difficulty in this country. The National Council for Combating Venereal Diseases has recently published a pronouncement against this proposal, based mainly on the statement that the chief evil is solicitation by "clandestine" rather than "regular" prostitutes. Since there are no "regular" prostitutes here, and the so-called "clandestines" are certainly "habituals," it is not easy to see what is meant by this verbal distinction. Nor does it seem impossible at this special juncture for police regulations to forbid at least the massing of any women at the railway stations on the arrival of the troop trains, as well as manifest "soliciting" in the streets.

If, however, nothing can be done in this direction (and it seems now unlikely that anything will be done) the method of prevention by the personal use of medicaments stands alone; and this should no longer be hindered by half-hearted support, or total opposition, in deference to so-called public opinion on the part of public authorities. Let any well-instructed medical practitioner, with a due knowledge of human physiology and psychology, seriously ask himself or herself the question whether moral and physical "education" on the one hand, or the use of really effectual preventives on the other, is the more likely means of checking venereal disease at its source—*as medical science strives to do in the case of all other infectious diseases*—and there can be no doubt whatever of what the answer would be.

In the *Times* of February 19 Dr. Helen Wilson, commenting on the newly proposed Criminal Law Amendment Bill, says: "While the masculine demand exists it is folly to think that the supply can be cut off." But what about the human race if the masculine demand did not exist?—I am, Sir, yours faithfully,

H. Bryan Donkin.

